# Screening of Bioactive Compounds from Endophytic Marine-Derived Fungi in Saudi Arabia: Antimicrobial and Anticancer Potential

**DOI:** 10.3390/life12081182

**Published:** 2022-08-03

**Authors:** Aisha M. H. Al-Rajhi, Abdullah Mashraqi, Mohamed A. Al Abboud, Abdel-Rahman M. Shater, Soad K. Al Jaouni, Samy Selim, Tarek M. Abdelghany

**Affiliations:** 1 Department of Biology, College of Science, Princess Nourah bint Abdulrahman University, Riyadh 11671, Saudi Arabia; 2 Biology Department, Faculty of Science, Jazan University, Jazan 82817, Saudi Arabia; mashraqi@jazanu.edu.sa (A.M.); mohalabboud@hotmail.com (M.A.A.A.); alhababi2010@gmail.com (A.-R.M.S.); 3 Biology Department, Faculty of Science, Thamar (Dhamar) University, Dhamar 00967, Yemen; 4 Department of Hematology/Oncology, Yousef Abdulatif Jameel Scientific Chair of Prophetic Medicine Application, Faculty of Medicine, King Abdulaziz University, Jeddah 21589, Saudi Arabia; saljaouni@kau.edu.sa; 5 Department of Clinical Laboratory Sciences, College of Applied Medical Sciences, Jouf University, Sakaka 72341, Saudi Arabia; 6 Botany and Microbiology Department, Faculty of Science, Al-Azhar University, Cairo 11884, Egypt

**Keywords:** endophytic, fungi, mangrove, antimicrobial, anticancer, *Penicillium rubens*

## Abstract

Nowadays, endophytic fungi represent a rich source of biological active compounds. In the current study, twelve endophytic fungal species were isolated from *Avicennia marina* leaves. From the isolates, *Aspergillus niger*, *Penicillium rubens* and *Alternaria alternata* recorded the highest isolation frequency (80%), relative density (12.5%) and antimicrobial activity. The antimicrobial and anticancer activities of *P. rubens* were more effective than those of *A. niger* and *A. alternata*; therefore, its identification was confirmed via the ITS rRNA gene. Filtrate extracts of *P. rubens*, *A. alternata* and *A. niger* were analyzed using GC-MS and showed different detected constituents, such as acetic acid ethyl ester, *N*-(4,6-Dimethyl-2-pyrimidinyl)-4-(4-nitrobenzylideneamino) benzenesulfonamide, 1,2-benzenedicarboxylic acid, hexadecanoic acid and octadecanoic acid. Filtrate extract of *P. rubens* exhibited the presence of more compounds than *A. alternata* and *A. niger*. *Bacillus subtilis*, *Staphylococcus* *aureus*, *Escherichia coli*, *Pseudomonas aeruginosa*, *Candida albicans* and *Aspergillus fumigatus* were more inhibited by *P. rubens* extract than *A. alternata* or *A. niger*, with inhibition zones of 27.2 mm, 22.21 mm, 26.26 mm, 27.33 mm, 28.25 mm and 8.5 mm, respectively. We observed negligible cytotoxicity of *P. rubens* extract against normal cells of human lung fibroblasts (WI-38 cell line), unlike *A. alternata* and *A. niger* extracts. Proliferation of prostate cancer (PC-3) was inhibited using *P. rubens* extract, exhibiting mortality levels of 75.91% and 76.2% at 200 µg/mL and 400 µg/mL of the extract. Molecular docking studies against the crystal structures of *C. albicans* (6TZ6) and the cryo-EM structure of *B. subtilis* (7CKQ) showed significant interactions with benzenedicarboxylic acid and *N*-(4,6-dimethyl-2-pyrimidinyl)-4-(4-nitrobenzylideneamino) benzenesulfonamide as a constituent of *P. rubens* extract. *N*-(4,6-dimethyl-2-pyrimidinyl)-4-(4-nitrobenzylideneamino) benzenesulfonamide had the highest scores of −6.04905 kcal/mol and −6.590 kcal/mol towards (6tz6) and (7CKQ), respectively.

## 1. Introduction

Microorganisms, particularly endophytes isolated from diverse marine habitats, have been extensively studied because they are considered a rich resource for pharmaceuticals and agrochemicals [1,2]. As remarked in existing studies, the marine microorganisms’ adaptation to their unique environments has led to the production of natural compounds with attractive biological activity [3,4]. Endophytes are microorganisms that dwell inside plants, such as fungi and bacteria. According to Pinheiro et al. [5], they colonize healthy parts of the plant at specific phases associated with plant life cycles, without any effects on plant health, including visible damage or the development of evident exterior structures [6]. Endophytic fungi have been found to be a rich source of new secondary metabolites with intriguing biological activity and a wide range of chemical compounds. These secondary metabolites have essential ecological roles as parasiticides and disease inhibitors, as well as other biological activities. As noted in some scientific papers, microorganisms associated with plants (endophytic fungi) in nature are widely acknowledged as some of the richest sources of novel beneficial metabolites for humans as well as plants [6].

A number of characteristics are associated with mangrove sediments, such as low pH, high salinity, strong reduction and nutrient richness, and they are are considered rich resources for microorganism colonization [7]. Overall, more diverse endophyte fungi were recorded on leaves compared with other plant parts. Vital biological utilities such as anticancer, antimicrobial, antioxidants and insecticidal properties have been documented from endophytic fungi of mangrove, particularly of leaf origin [8]. There is a growing need for safe antimicrobial agents as well as antibiotics to treat microbial infections in humans and animals. Antimicrobial activities are associated with a large number of extracts or products from fungi. *Penicillium* sp. is the main filament fungus for antibiotics production [9,10,11]. A number of species of *Penicillium* are recognized to produce secondary metabolites, among which *P. chrysogenum* [12] exhibited antimicrobial activities against *Pseudomonas aeruginosa*, *Staphylococcus aureus*, *Bacillus cereus* and *Fusarium oxysporum* and against amoeba *Acanthamoeba polyphaga*. Excellent bioactivity of *Penicillium citrinum* was reported against *Klebsiella pneumoniae* and *Staphylococcus aureus* [13]. The ability of the Ascomycota group, which contains *Penicillium* spp., for the creation of antimicrobial metabolites is greater compared to other groups [11].

According to some recent literature, *Penicillium* spp. still represents the main producer of various secondary metabolites [14]. Not only antimicrobial activities but other biological activities have been reported using *Penicillium* spp. secondary metabolites. Previous studies indicated that several species of *Penicillium* can inhabit various environments with different conditions, including indoor air, soils, foods and drinks [15,16]. Eleven of ninety-five endophytic fungi exhibited antibacterial activity, with the greatest antibacterial activity against *Staphylococcus aureus* and *Streptococcus pyogenes*, which resulted from *Penicillium commune*, *P. glabrum*, *Aspergillus ochraceus* and *Gibberella baccata* [6].

Molecular docking is a technique applied in the field of molecular modeling that forecasts the preferred orientation of one molecule to another when they are linked together to form a stable complex [17]. By inserting a molecule (ligand) into the preferred binding site of the specific target region of the DNA/protein (receptor), the information obtained can be used to indicate the binding energy, free energy and stability of complexes [18]. Docking can be applied to predict where and in which relative orientation a ligand binds to a protein (i.e., binding mode or position). With this knowledge, more potent and targeted analogues may be generated. Docking combined with a scoring function can be applied to quickly screen large databases of potential drugs in silico to identify molecules that are likely to bind to protein targets of interest [19]. 

Endophytic fungi produce a variety of secondary metabolites with potential medical application and hence provide an opportunity for the discovery of new antibiotics. As noted in the Introduction, endophytic—particularly marine-derived—fungi are considered a promising agent for the creation of novel compounds with medicinal applications. Therefore, the search for endophytic fungi from marine habitats and the evaluation of their biological activities, including antimicrobial and anticancer, were the main goals of the current study. 

## 2. Materials and Methods

### 2.1. Source of Endophytic Fungi

Endophytic fungi were isolated from mangrove plants (*Avicennia marina*) that were propagated in the Jazan region, Saudi Arabia, situated along the Red Sea, located at 16°47′00.7″ N latitude and 42°40′30.8″ E.

### 2.2. Endophytic Fungi Isolation and Cultivation

The collected plant leaves of *A. marina* were washed several times (5 times) using running tap water to remove any debris such as dust or the waste of birds, and then dipped in ethanol (70%) for 1–2 min. Under aseptic conditions, the leaves were ruptured into small segments (2 mm) and then dipped in NaOCl (4%) for 1 min. The small segments were rinsed in sterile distilled water 5 times, followed by drying via sterile filter paper [20]. The dried segments were placed on fungal growth agar medium plates that contained 15 g/L of malt extract, 10 g/L of artificial sea salt, 20 g/L of bacto agar, 1 L of sterile distilled water and 0.2 g/L of chloramphenicol to prevent bacterial growth. Medium pH was adjusted to 7.7 according to the pH analysis of sea water. At 30 °C, the plates were incubated for 8 days. The appeared fungal hyphae tips from the segments of leaves were sub-cultured several times on the same growth medium to obtain pure fungal colonies.

The isolation frequency (IF) and relative density (Rd) of species were recorded as follows:

$$\mathrm{IF}\;\left(\%\right)=\frac{\mathrm{No}.\;\mathrm{of}\;\mathrm{occurrence}\;\mathrm{samples}\;\mathrm{of}\;a\;\mathrm{species}}{\mathrm{Total}\;\mathrm{no}.\;\mathrm{of}\;\mathrm{samples}}\times 100$$

$$\mathrm{Rd}\;\left(\%\right)=\frac{\mathrm{No}.\;\mathrm{of}\;\mathrm{occurrence}\;\mathrm{samples}\;\mathrm{of}\;a\;\mathrm{species}}{\mathrm{Total}\;\mathrm{no}.\;\mathrm{of}\;\mathrm{isolated}\;\mathrm{fungi}}\times 100$$

### 2.3. Identification of Fungal Isolates

To identify the fungal isolates, the pure fungal cultures were re-cultivated on different media (potato dextrose agar, malt extract and Czapek Dox agar were provided by Sigma-Aldrich, St. Louis, MO, USA) to assess the growth rate, colony color and form, margin characteristics and elevation. At the same time, the fungal colonies were microscopically examined to visualize the shape and diameters of fungal mycelia, conidiophores, conidiospores, chlamydospores, sexual spores and fruiting bodies [21,22,23,24,25]. The fungus’ potent antimicrobial activity was identified molecularly as follows. DNA was extracted from 100 mg of fungus mycelia (collected from fungus growing on broth medium). The 18S ribosomal DNA (rDNA) internal transcribed spacer (ITS) region was amplified via polymerase chain reaction (PCR) using the Quick-DNA Fungal Microprep Kit (Zymo research; D6007; Irvine, CA, USA) with the following primers: ITS1-forward (5′-TCCGTAGGTGAACCTGCGG-3′) ITS4-reverse (5′-TCCTCCGCTTATTGATATGC-3′) [26,27], at Sigma Scientific Services Company, Cairo, Egypt. The total volume of PCR reaction containing the extracted genomic DNA (10 µL) included 1 µL of each primer (forward primer 18S rRNA and reverse primer 18S rRNA) and 1 µL of nuclease, and 25 µL of Maxima^®^ 2× Hot Start PCR Master Mix was prepared to perform the reaction of PCR. PCR amplification was performed at the following temperature cycles: a first step of 2 min at 94 °C for the first initial step, followed by 40 cycles at 94 °C for 60 s, then at 52 °C for 90 s, and for 2 min at 72 °C, and a final cycle at 72° for 7 min.

### 2.4. Preparation of Marine Fungal Extract

After the incubation period, 100 mL of the metabolized medium free of fungal mycelia was filtrated via filter paper (Whatman No. 1 filter paper). At low pressure (8 × 10^3^ Pa), the fungal filtrate was evaporated to obtain the extract, which was subsequently extracted with methanol and then concentrated to obtain a crude extract for further study, including gas chromatography–mass spectrometry (GC-MS) analysis and biological activities. 

### 2.5. GC-MS Analysis of Fungal Filtrate Extract

The fungal extract was analyzed using GC (THERMO Scientific Corp., Waltham, MA, USA) joined with MS (ISQ Single Quadrupole Mass Spectrometer, Waltham, MA, USA). Fungal extract (1 µL) was injected into the chromatography column (30 m × 0.32 mm × 0.25 μm) via the Autosampler AS1300. Temperature cycling was programmed at 60 °C for initial analysis, and then increased to 240 °C. Subsequently, it was increased gradually to 290 °C for 2 min. Helium with the highest purity was utilized as a carrier at a rate of 1 mL/min. The electron ionization mass of the spectra was collected in full scan mode at a range of *m*/*z* 40–1000 via electron energy of 70 eV as desired. Then, via calculation of the retention time (RT) and mass spectra of the discovered compounds, they were identified by comparison to the mass spectra logged at the library of the National Institute of Standards and Technology [28].

### 2.6. Disk Diffusion Method and MIC Detection for Antimicrobial Activity of Fungal Extract

The antimicrobial activity of the fungal extract was assayed against 4 species of bacteria, including *Bacillus subtilis, Escherichia coli,* *Staphylococcus aureus* and *Pseudomonas aeruginosa* (provided by Hospitals of Ain Shams University in Egypt), as a source of the tested fungi and two fungi including *Candida albicans* and *Aspergillus fumigatus* (provided by Assiut University Mycological Centre in Egypt). A disk of filter paper (6 mm) was loaded with 100 µL of fungal filtrate extract and then pasted on a nutrient agar medium surface inoculated with tested bacteria, while potato dextrose agar medium was used for tested fungi. Then, the plates were kept in a refrigerator at 3 °C for 20 min for suitable extract diffusion. After this, the plates were transferred to an incubator (adjusted at 28 °C for 3 days required for fungi and 37 °C for 24 h required for bacterial growth). The visualized clear zone around the disc loaded with fungal filtrate extract was measured (mm). Control discs were loaded with antibiotic gentamycin as an antibacterial agent and ketoconazole as an antifungal agent [29]. A microdilution protocol was applied to determine the minimum inhibitory concentration (MIC) of fungal filtrate extract. Different dilutions of the extract that were dissolved in dimethylsulfoxide (DMSO) were prepared through the production of serial dilutions. Suspensions of bacteria and fungi inocula were prepared (2 × 10^8^ and 2 × 10^6^ colony-forming units/mL of bacteria and fungi, respectively) and inoculated (100 μL) in macro-dilution tubes containing the fungal filtrate extract. At 37 °C for 18 h (required for bacteria) or for 24 h (required for yeast), the macro-dilution tubes were incubated, followed by measuring the OD at 600 nm.

### 2.7. Cytotoxicity Assay against Cancer Cells 

The used culture cells were prostate cancer cells (PC-3) that were provided by Nawah Scientific Inc. (Mokatam, Cairo, Egypt). DMEM medium, amended with streptomycin (100 mg/mL), penicillin (100 units/mL) and heat-inactivated fetal bovine serum (10%) and humidified, was used to maintain the cells under CO_2_ (5% *v*/*v*) and at 37 °C. The SRB assay was applied to determine the effect of the fungal extract on the PC-3 viability. Suspensions (100 μL) of PC-3 containing 5 × 10^3^ cells were placed in 96-well plates, followed by incubation for 24 h. Different concentrations of the fungal extract were added to cell medium; 100 μL of medium containing the extract was added to the cultivated cells and incubated for 3 days. Then, the treated cells were fixed via 150 μL of 10% TCA instead of media, followed by incubation for 1 h at 4 °C. After this, the solution of TCA was removed, followed by washing the cells using distilled water 5 times. Aliquots of 70 μL of 0.4% *w*/*v* SRB solution were added to PC-3, and then incubated for 10 min in a dark area at 25 °C. With 1% acetic acid, the plates were washed 4 times and then allowed to dry in air for 12 h; subsequently, 150 μL of TRIS (10 mM), required to dissolve the protein-bound SRB stain, was added; at this point, using the BMG LABTECH^®^-FLUOstar Omega microplate reader (Ortenberg, Germany), the absorbance was recorded at 540 nm [30]. 

### 2.8. Cytotoxicity Assay against Normal Cells

The used culture cells were the WI-38 cell line (human lung fibroblast normal cells) that were provided by Nawah Scientific Inc. (Mokatam, Cairo, Egypt). Cells were propagated in Dulbecco’s modified Eagle’s medium (DMEM) amended with 10% fetal bovine serum (FBS) at 37 °C in a humidified incubator (5% CO_2_). A suspension (100 μL) of the WI-38 cell line (5 × 10^3^ cell/mL) was plated in microtiter plates (96-well), followed by incubation for 18 h. Subsequently, different doses of filtrate fungal extract were loaded onto each used well and the incubation period was completed, lasting 2 days. To each well, the prepared solution (20 μL) of 3-(4,5-dimethylthiazol-2-yl)-2,5-diphenyltetrazolium bromide (5 mg/mL) was added, followed by incubation for 4 h. The absorbance was recorded at 540 nm to estimate the cytotoxicity activity [30].

### 2.9. Molecular Docking Studies with MOE

As a structural biology method, molecular docking was used to evaluate the interactions between pairs of molecules, typically a receptor and a ligand. Molecular docking modeling was performed using the Molecular Modeling Environment (MOE) (MOE 2019.0102 program). The crystal structure of *C. albicans* (6TZ6) and the cryo-EM structure of *B. subtilis* (7CKQ) were downloaded from the Protein Data Bank (https://www.rcsb.org/pdb/ (accessed on 19 July 2022)). The native compounds were prepared prior to the docking experiment by minimizing their energy at the B3LYP/631G (d) level of theory. Partial charges were calculated by Gasteiger’s method. The last structure was acquired after 3D protonation and the correction process. The bound water, ligands and cofactors were removed from the protein. The MOE site finder generated the active binding sites to create the dummy sites as the binding pocket. Using the triangular matching docking method, the target molecules were docked into the protein’s active site, and thirty conformations of the title compounds and protein complex were created with a docking score (S kcal/mol). The docking protocol predicted the best five conformations as present in the crystal structure with a better RMSD value. The complexes were analyzed for interactions and their 3D images were taken by using a visualizing tool, PyMol (The PyMOL Molecular Graphics System, Version 1.2r3pre, Schrödinger, LLC, New York, NY, USA).

Schrödinger Release 2022-2: BioLuminate, Schrödinger, LLC, New York, NY, 2021. Additionally, the results in the co-crystal ligand position before and after amendment, respectively, were compared pose-with-pose using the RMSD and RMSD-refine fields [31].

The following definitions were used in the molecular docking studies: final score (S), which is the score of the last stage that was not set to zero; the root mean square deviation (rmsd) of the pose, in Å, from the original ligand—this field is present if the site definition was identical to the ligand definition; rmsd_refine, meaning the root mean square deviation among the pose before refinement and the pose after refinement; E_conf meaning the energy of the conformer—if there is a refinement stage, this is the energy estimated at the end of the refinement. Annotation was performed for force field refinement; by default, this energy is estimated with the solvation option set to Born. We also included E_place, meaning the score from the placement stage; E_score 1 and E_score 2, meaning the scores from rescoring stages 1 and 2; and E_refine, meaning the score from the refinement stage, estimated to be the whole of the van der Waals electrostatics and solvation energies under the generalized Born solvation model. 

### 2.10. Statistical Analysis 

All experimental results were realized in triplicate. The standard deviation (SD) and variance were calculated via SPSS ver. 22.0 software (version 14, IBM Corp., Armonk, NY, USA). 

## 3. Results and Discussion 

### 3.1. Endophytic Fungi Isolates

**Figure 1:**
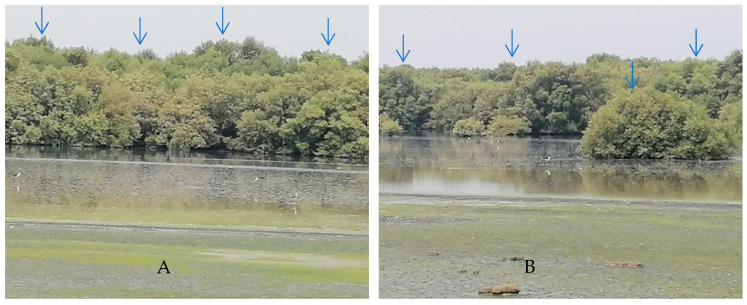

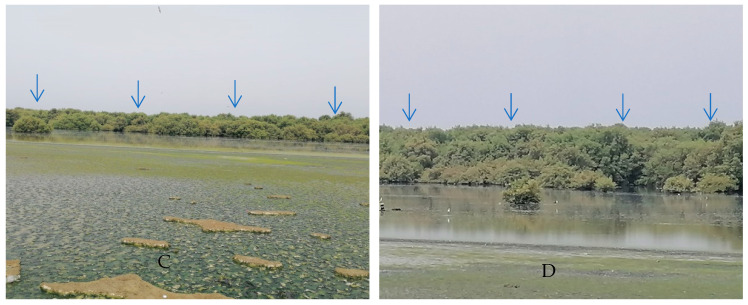
Four sites (**A**–**D**) with mangrove clumps (blue arrows) in Jazan region of Saudi Arabia as a source of endophytic fungi isolation.

**Figure 2:**
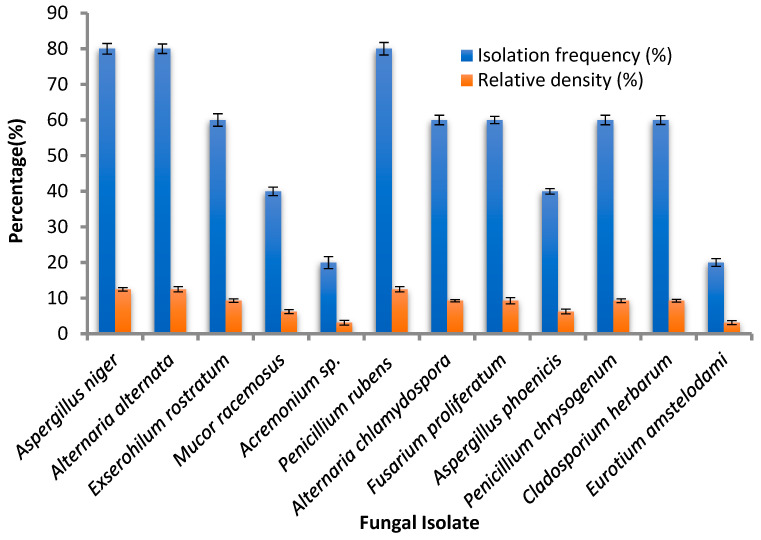
Isolation frequency and relative density % of the isolated endophytic fungi. Error bars represent the standard deviations.

**Figure 3:**
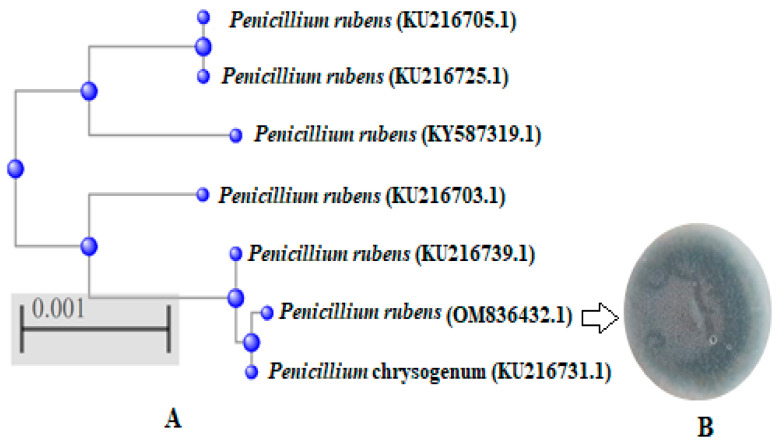
Identification of *P. rubens* (OM836432.1) with neighbor joining phylogenetic tree (**A**) and its colony (**B**). sharper.

Thirty fungal isolates, belonging to twelve fungal species, were isolated from leaf segments of *Avicennia marina* collected from four sites (A, B, C and D) in the same region that was characterized by mangrove clumps (Figure 1A–D) in Jazan city, Saudi Arabia. The fungal isolates belonged to *Aspergillus niger*, *Alternaria alternata*, *Exserohilum rostratum*, *Mucor racemosus*, *Acremonium* sp., *Penicillium rubens*, *Alternaria chlamydospora*, *Fusarium proliferatum*, *Aspergillus phoenicis*, *Penicillium chrysogenum*, *Cladosporium herbarum* and *Eurotium amstelodami* with different IF (%) and Rd (%). *A. niger*, *P. rubens* and *A. alternata* occurred with the highest isolation frequency (80%) and relative density (12.5%), while other isolates showed the lowest IF (20%) and Rd (3.12%), specifically *E. amstelodami* and *Acremonium* sp. Our results were partially in line with the obtained results of Hamed et al. [32], who identified various endophyte fungi of marine habitats that belonged to eight different genera of *Cladosporium*, *Epicoccum*, *Alternaria*, *Aspergillus*, *Byssochlamys*, *Talaromyces Penicillium* and *Sarocladium*. The dominant species *P. rubens*, *A. alternata* and *A. niger* in the current study were identified to the level of species, also as mentioned by Hamed et al. [32]. Some previous reports indicated that the fungal diversity is influenced by the availability and sources of nutrients, as well as the physicochemical factors of the coastal ecosystem [33]. Therefore, in another study, *Acremonium curvulum* was the most frequently found endophyte (9.5%), followed by *Aspergillus ochraceus*, isolated from *Bauhinia forficata* [6]. The three fungal isolates with the highest isolation frequency (Figure 2) were assessed for antimicrobial activity against different bacteria and fungi. The identification of the fungus with the highest antimicrobial activity was confirmed with molecular identification via the ITS rRNA gene. Through the alignment search tool available at https://blast.ncbi.nlm.nih.gov (accessed on 19 July 2022), the value of ITS rRNA homology for the isolated strain exhibited resemblance 99.12% and query cover 100% with *P. rubens* (OM836432.1) as existing in the phylogenetic tree (Figure 3); the sequence was recorded in GenBank with accession number OM836434.1.

### 3.2. GC-MS Analysis of Fungal Extract 

**Figure 4:**
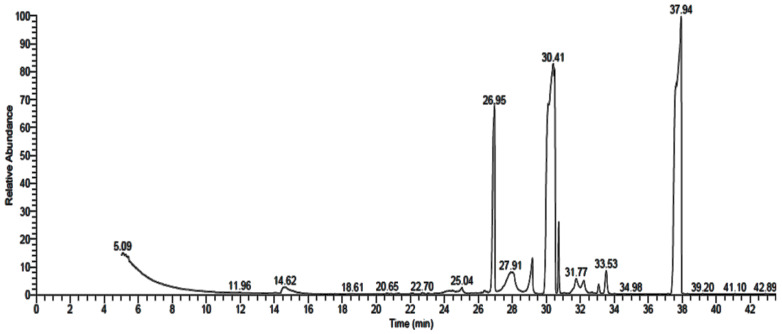
GC-MS analysis chromatogram of metabolized medium extract of *P. rubens*.

**Figure 5:**
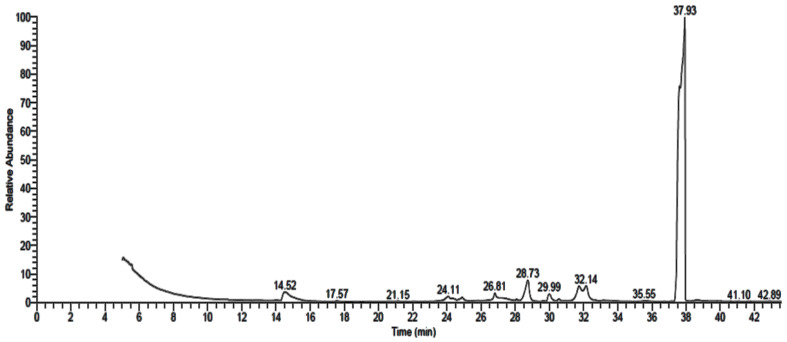
GC-MS analysis chromatogram of metabolized medium extract of *A. alternata*.

**Figure 6:**
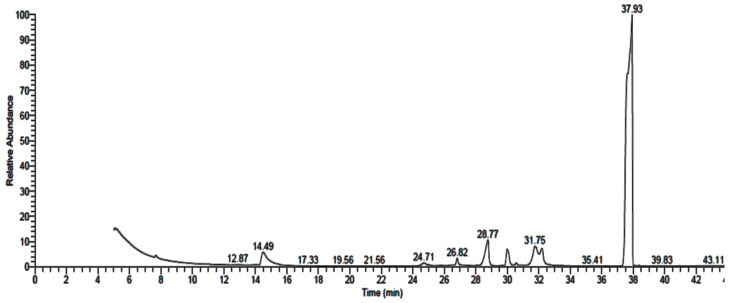
GC-MS analysis chromatogram of metabolized medium extract of *A. niger*.

GC-MS analysis of *P. rubens*, *A. alternata* and *A. niger* growth media extracts showed the existence of 16 (Table 1 and Figure 4), 9 (Table 2 and Figure 5) and 10 (Table 3 and Figure 6) compounds with different retention times and areas (%). Acetic acid ethyl ester, *N*-(4,6-dimethyl-2-pyrimidinyl)-4-(4-nitrobenzylideneamino) benzenesulfonamide, 1,2-benzenedicarboxylic acid, hexadecanoic acid and octadecanoic acid were detected in the filtrate extracts of the three fungal isolates. On the other hand, some compounds, such as tetradecanoic acid, tributyl acetylcitrate and propiolic acid, 3-(1-hydroxy-2-isopropyl-5-methylc yclohexyl)-, ethyl ester, were identified in the filtrate extract of *P. rubens* only. At the same time, oxiraneundecanoic acid, 3-pentyl-, methyl ester, cis and 9,12-octadecadienoyl chloride, (Z,Z) were identified in the filtrate extract of *A. alternata*. Secondary metabolites, which are created by marine-derived fungi, are potential sources of unique bioactive compounds. Multiple studies have focused on the isolation of endophytic fungi from diverse geographical and environmental regions to evaluate their productivity of various compounds with multiple uses in medicinal applications, such as anticancer, antiviral, antimicrobial and antioxidant activities [34,35,36]. According to some studies, antimicrobial, antioxidant, anticancer and anti-inflammatory activities were attributed to the compounds—or their derivatives—that were found in the current study, such as n-hexadecanoic acid, 9,12-octadecandionoic acid, 11-octadecenoic acid and methyl ester [37]. Methyl stearate, which was identified in *P. rubens* extract (Table 1), has been applied as an antifungal, intestinal lipid regulating, nematicidal and anti-inflammatory agent [38]. Hexadecanoic acid and methyl ester (area 16.41%), found in the current study, previously exhibited antibacterial activity against definite Gr + ve and Gr − ve bacteria [39]. Results of GC-MS analysis of *P. crustosum* filtrate extract revealed that hexadecanoic acid was the main detected constituent [1]. The antibacterial and antioxidant activities of hexadecanoic acid have been documented [1,40].

### 3.3. Antimicrobial Activity of Fungal Extract

**Figure 7:**
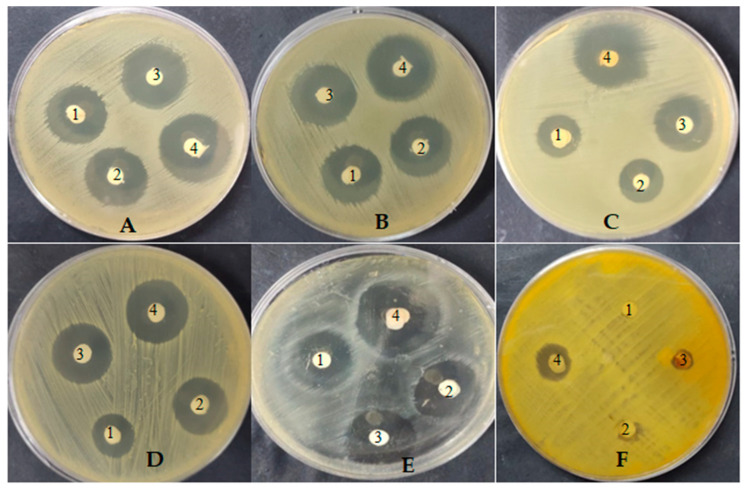
Antimicrobial activity of *A. alternata* (1), *A. niger* (2) and *P. rubens* (4) extracts and control (3) against *B. subtilis* (**A**), *E. coli* (**B**), *P. aeruginosa* (**C**), *S. aureus* (**D**), *C. albicans* (**E**) and *A. fumigatus* (**F**).

Antimicrobial activity of *P. rubens*, *A. alternata* and *A. niger* filtrate extracts was recorded against various microorganisms, but with different levels of growth inhibition (Table 4 and Figure 7). The highest activity was visualized using *P. rubens* filtrate extract against all tested bacteria, namely *B. subtilis* (inhibition zone was 27.2 mm), *S.aureus* (inhibition zone was 22.21 mm), *E. coli* (inhibition zone was 26.26 mm) and *P. aeruginosa* (inhibition zone was 27.33 mm), as well as fungi, namely *C. albicans* (inhibition zone was 28.25 mm) and *A. fumigatus* (inhibition zone was 8.5 mm). Meanwhile, *A. alternata* and *A. niger* exhibited lower antibacterial activity than *P. rubens*. At the same time, *A. alternata* and *A. niger* did not show any antifungal activity against *C. albicans* and *A. fumigatus*. Several studies have been performed to explore new antimicrobial compounds derived from endophytic fungi. Bezerra et al. [6] tested the antimicrobial activity of some isolates of endophytic fungi, and they found that 34.3% of the fungal isolates exhibited activity, particularly *P. glabrum*, *P. commune*, *Aspergillus ochraceus* and *Gibberella baccata.* The MIC of *P. rubens* filtrate extract only was evaluated against tested organisms, with MIC 19.50 µg/mL, 22.30 µg/mL, 21.62 µg/mL, 19.70 µg/mL, 31.2 µg/mL and 39.50 µg/mL for *B. subtilis*, *S. aureus*, *E. coli*, *P. aeruginosa* *C. albicans* and *A. fumigatus*, respectively (Table 4).

Methyl stearate was among the detected compounds in *P. rubens* filtrate extract; according to a previous study [37], it possesses antifungal activity. Moreover, in agreement with Davoodbasha et al. (2018), Gr + ve and Gr − ve bacteria were inhibited by hexadecanoic acid methyl ester, which was detected in the filtrate extract of *P. rubens*. Some compounds—for example, *N*-(4,6-dimethyl-2-pyrimidinyl)-4-(4-nitrobenzylideneamino) benzenesulfonamide and 1,2-benzenedicarboxylic acid, were included in the filtrate extracts of *P. rubens*, *A. alternata* and *A. niger*; the antimicrobial activity may due to the presence of such compounds. Our results are in line with the findings of Basheer [41], who found that most of the endophytic fungi isolated from *A. marina* (red sea in Egypt) had antimicrobial activity but with different levels of microbial growth inhibition. Moreover, from Malaysian mangrove *Rhizophora mucronata*, various endophytic fungi were isolated and exhibited bactericidal effects against *E. coli*, *P. aeruginosa*, *S. aureus* and *B. subtilis* [8]. In another study, extracts of marine-derived Turkish fungi such as *A. alternata* and *A. niger* exhibited antimicrobial activity against *S. aureus*, *S. epidermidis*, *B. subtilis*, *E. coli*, *P. aeruginosa*, *Klebsiella pneumoniae*, *C. albicans* and *C. parapsilosis*, but *P. rubens* exhibited low and narrow-spectrum antimicrobial activity against *P. aeruginosa*, *B. subtilis* and *E. coli* only [42].

### 3.4. Cytotoxicity of Fungal Extract

**Figure 8:**
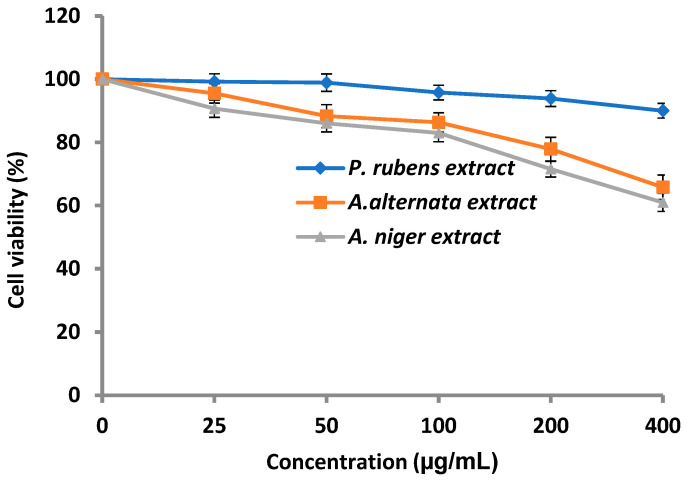
Cytotoxicity of *P. rubens*, *A. alternata* and *A. niger* against normal cells of human lung fibroblasts. Error bars represent the standard deviations.

The cytotoxicity of *P. rubens*, *A. alternata* and *A. niger* extracts was evaluated against normal cells of human lung fibroblasts (WI-38 cell line) (Figure 8). The results indicated that *P. rubens* extract showed negligible cytotoxicity at concentrations up to 100 µg/mL and weak cytotoxicity at concentrations up to 400 µg/mL and reached 9.09% mortality, suggesting the possibility of further anticancer activity, as well as other pharmaceutical applications. On the other hand, the cytotoxicity of *A. alternata* and *A. niger* extracts was more evident than that of *P. rubens* extract, particularly at 400 µg/mL.

**Figure 9:**
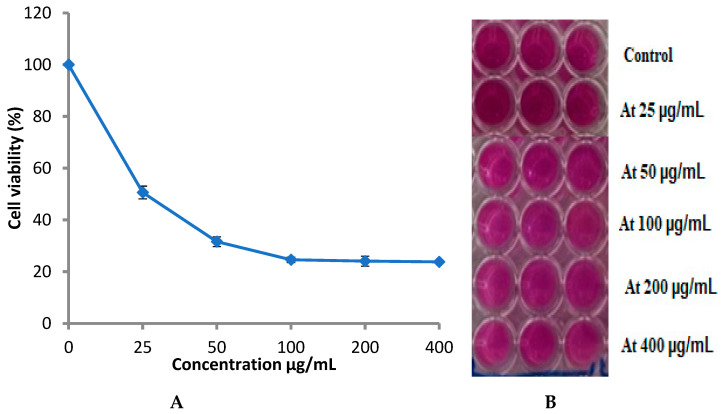
Cytotoxicity of *P. rubens* against prostate cancer cell line (**A**), the 96-well micro plate used in cytotoxicity assay at different concentrations (**B**). Error bars represent the standard deviations.

Therefore, the extract of *P. rubens* was tested against prostate cancer (PC-3) proliferation. The anticancer activity test showed that *P. rubens* extract exhibited inhibitory activity towards PC-3 proliferation in a dose-dependent manner (Figure 9), where the cell viability decreased with the increase in the extract concentration. At 25 µg/mL, cell viability was 50.60%, while at 200 µg/mL and 400 µg/mL, the cell viability was 24.09% and 23.80%, reflecting mortality levels of 75.91% and 76.2%, respectively. Hence, the obtained findings also indicated that the endophytic fungus *P. rubens*, of marine habitats, has acceptable inhibitory activity for PC-3. *A. niger* and *P. rubens* displayed the highest anticancer activity against a colon cancer cell line (HCT-116) [42]. In a previous study, inhibition of HL-60 and A-549 cell line proliferation may have been to chloctanspirone A, as identified by Bladt et al. [43] in *P. rubens* extract.

### 3.5. Molecular Docking Studies

**Figure 10:**
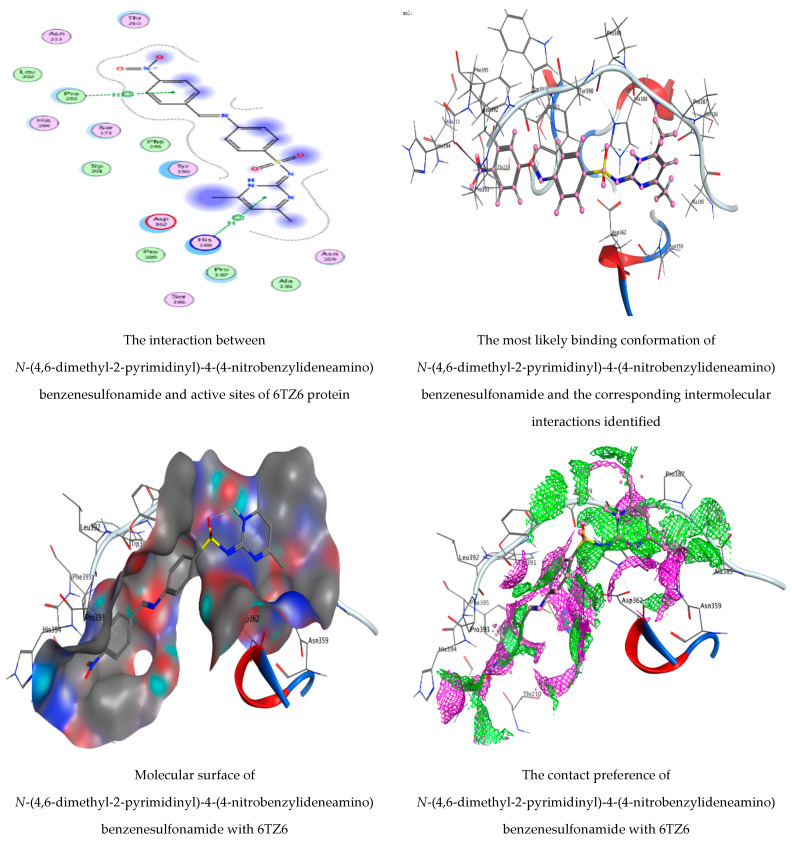

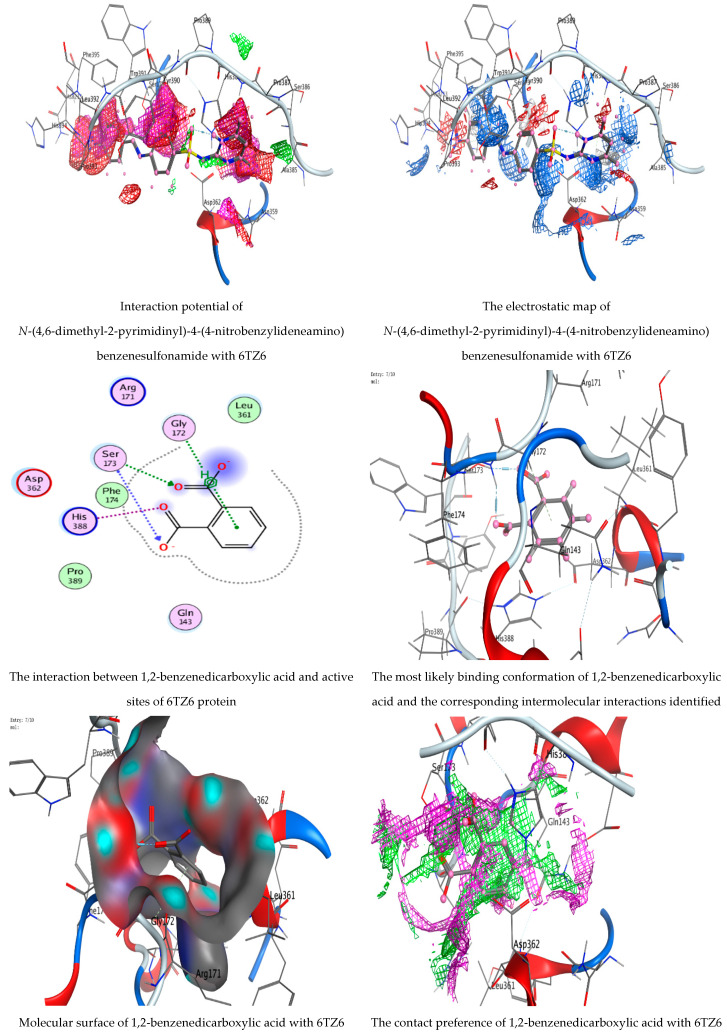

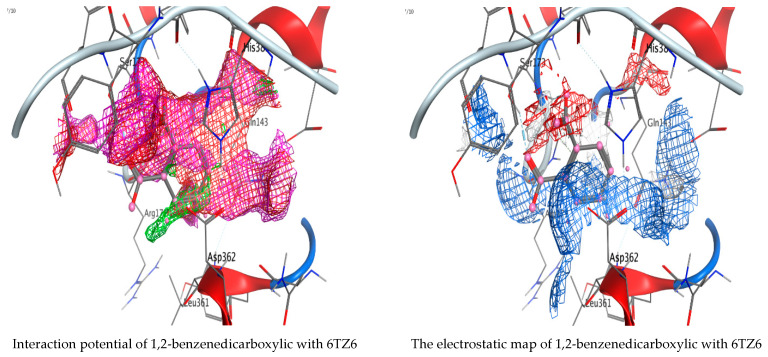
Molecular docking process of *N*-(4,6-dimethyl-2-pyrimidinyl)-4-(4-nitrobenzylideneamino) benzenesulfonamide and 1,2-benzenedicarboxylic acid with 6TZ6 protein.

**Figure 11:**
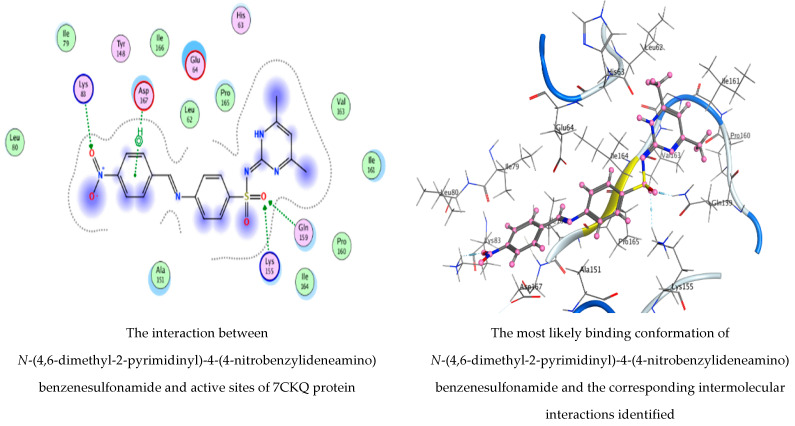

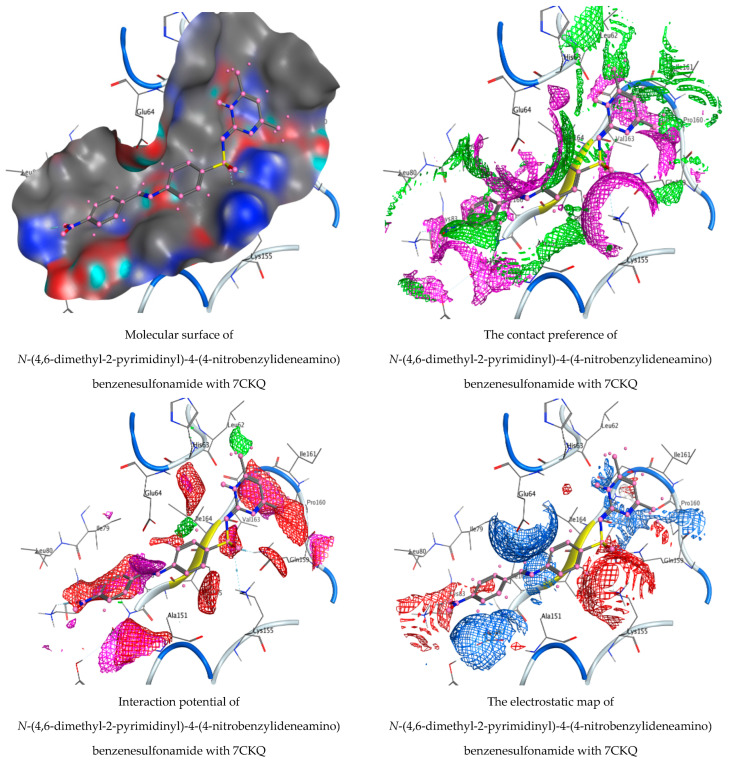

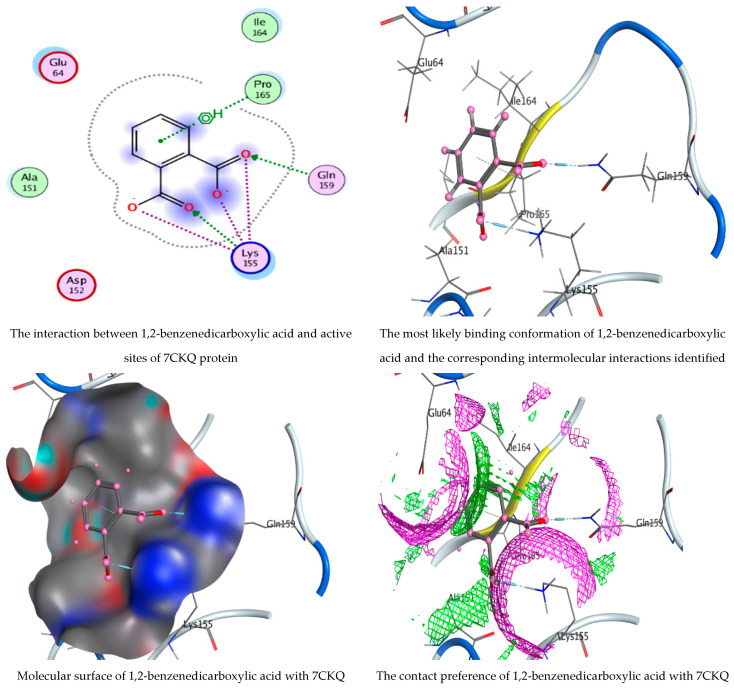

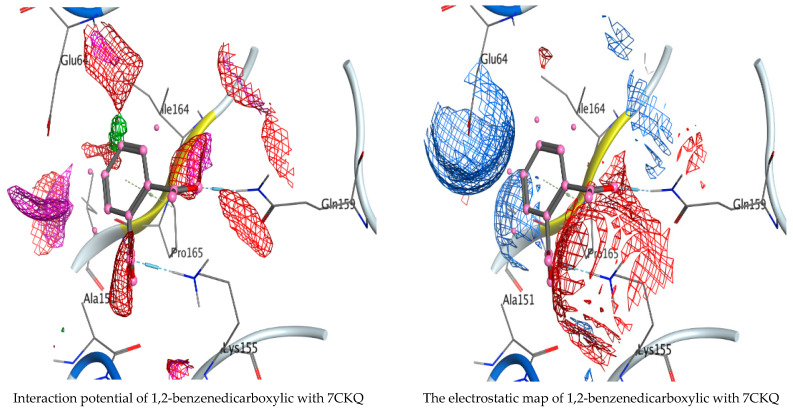
Molecular docking process of *N*-(4,6-dimethyl-2-pyrimidinyl)-4-(4-nitrobenzylideneamino) benzenesulfonamide and 1,2-benzenedicarboxylic acid with 7CKQ protein.

*N*-(4,6-Dimethyl-2-pyrimidinyl)-4-(4-nitrobenzylideneamino) benzenesulfonamide and 1,2-benzenedicarboxylic acid, as detected in the filtrate extract of *P.rubens*, were docked against the crystal structure of *C. albicans* (PDB = 6TZ6) and the cryo-EM structure of *B. subtilis* (PDB = 7CKQ) to elucidate the antimicrobial activity of the fungal filtrate extract. We noticed that *N*-(4,6-dimethyl-2-pyrimidinyl)-4-(4-nitrobenzylideneamino) benzenesulfonamide had the lowest binding energy score with both 6TZ6 and 7CKQ proteins revealing the highest activity at −6.04905 kcal/mol and −6.590 kcal/mol, respectively. On the contrary, 1,2-benzenedicarboxylic acid showed docking scores of −4.74927 kcal/mol and −3.97767 kcal/mol for 6TZ6 and 7CKQ, respectively. The ranking poses generated by the scoring functions are given in Table 5 and Table 6. A list of hydrogen bonds between compounds with coenzymes of the chosen proteins is, respectively, presented in Table 7 and Table 8. Figure 10 and Figure 11 show, respectively, the best-fitting poses adopted by the compounds docked into proteins 6TZ6 and 7CKQ. In the case of *N*-(4,6-dimethyl-2-pyrimidinyl)-4-(4-nitrobenzylideneamino) benzenesulfonamide, we observed an interaction with 6TZ6 receptors via the 6-ring of the molecule and HIS 388, PRO 393 amino acid residues. Moreover, *N*-(4,6-dimethyl-2-pyrimidinyl)-4-(4-nitrobenzylideneamino) benzenesulfonamide interacted with 7CKQ by accepting the H atom of O 36 and O 45 through LYS 83, LYS 155 and GLN 159 amino acid receptors, in addition to a hydrogen bond between the aromatic ring of the molecule and the ASP 167 amino acid residue. On the other hand, the interaction of 1,2-benzenedicarboxylic acid with 6TZ6 formed two accepting hydrogen atoms between O 14 and O 16 in the ligand and the SER 173 amino acid residue, in addition to the hydrogen connection between the molecule’s aromatic ring and the GLY 172 amino acid in the receptor. Meanwhile, 7CKQ interacted with 1,2-benzenedicarboxylic acid through hydrogen bonding between two oxygen atoms of the compound towards the GLN 159 and LYS 155 amino acid residues. In the current study, *N*-(4,6-dimethyl-2-pyrimidinyl)-4-(4-nitrobenzylideneamino) benzenesulfonamide and 1,2-benzenedicarboxylic acid showed acceptable RMSD (the root mean square deviation of the pose, in Å, from the original ligand) values. The biological activity of a number of natural compounds has been documented with molecular docking studies—for example, the efficacy of chlorogenic acid on human coronavirus (HCoV 229E) and *Proteus vulgaris* [31]; *Aloe vera* gel based on chitosan nanoparticles against *Helicobacter pylori* [44]; and the efficacy of neophytadiene, luteolin, chrysoeriol and kaempferol against *P. aeruginosa*, *E. coli*, human prostate and breast-cancer-associated proteins, respectively [45].

## 4. Conclusions

*A. marina* leaves are rich in different and various endophytic fungi. *P. rubens* exhibited good antimicrobial and anticancer activities compared with the other isolates. The extract of *P. rubens* was more effective than other fungal extracts against the tested microorganisms due to the presence of a number of compounds that were detected in GC-MS analysis. Using molecular docking modeling, we discovered the effectiveness of the studied compounds against *C. albicans* and *B. subtilis*, which could motivate the design and synthesis of more antibacterial agents. Finally, the current article may help in our understanding of the fungal diversity of a number of marine environments with diverse biological utilities.

## Figures and Tables

**Table 1:** GC-MS analysis of metabolized medium extract of *Penicillium rubens*.

Detected Constituent	RT *	Area (%)	MF *	MW *
Acetic acid ethyl ester	5.43	0.27	C_4_H_8_O_2_	88
*N*-(4,6-Dimethyl-2-pyrimidinyl)-4-(4-nitrobenzylideneamino)benzenesulfonamide	14.55	0.87	C_19_H_17_N_5_O_4_S	411
Tetradecanoic acid	25.04	0.27	C_14_H_28_O_2_	228
Hexadecanoic acid, methyl ester	26.95	16.41	C_17_H_34_O_2_	270
Propiolic acid, 3-(1-hydroxy-2-isopropyl-5-methylc yclohexyl)-, ethyl ester	27.91	2.57	C_15_H_24_O_3_	252
(1,5,5,8-Tetramethyl-bicyclo [4.2.1]non-9-yl)-acetic acid	28.04	0.94	C_15_H_26_O_2_	238
Hexadecanoic acid	29.19	2.45	C_16_H_32_O_2_	256
9,12-Octadecadienoic acid (Z,Z)-, methyl ester	30.05	8.10	C_19_H_34_O_2_	294
9-Octadecenoic acid (Z)-, methyl ester	30.49	29.10	C_19_H_36_O_2_	296
Methyl stearate	30.74	3.34	C_19_H_38_O_2_	298
9,12-Octadecadienoic acid (Z,Z)-	31.76	0.86	C_18_H_32_O_2_	280
Octadecanoic acid	32.21	0.73	C_18_H_36_O_2_	284
Tributyl acetylcitrate	33.09	0.65	C_20_H_34_O_8_	402
8,11-Eicosadienoic acid, methyl ester	33.53	1.81	C_21_H_38_O_2_	322
Bis(2-ethylhexyl) phthalate	37.57	9.85	C_24_H_38_O_4_	390
1,2-Benzenedicarboxylic Acid	37.94	21.77	C_24_H_38_O_4_	390

* Note: Retention time (RT); molecular formula (MF); molecular weight (MW).

**Table 2:** GC-MS analysis of metabolized medium extract of *A. alternata*.

Detected Constituent	RT *	Area (%)	MF *	MW *
Acetic acid ethyl ester	5.57	0.59	C_4_H_8_O_2_	88
*N*-(4,6-Dimethyl-2-pyrimidinyl)-4-(4-nitrobenzylideneamino)benzenesulfonamide	14.46	3.25	C_19_H_17_N_5_O_4_S	411
Oxiraneundecanoic acid, 3-pentyl-, methyl ester, cis	26.80	0.97	C_19_H_36_O_3_	312
Hexadecanoic acid	28.74	4.07	C_16_H_32_O_2_	256
9,12-Octadecadienoyl chloride, (Z,Z)	29.98	1.82	C_18_H_31_ClO	298
9-Octadecenoic acid (Z)-	31.73	1.94	C_18_H_34_O_2_	282
Octadecanoic acid	32.17	1.80	C_18_H_36_O_2_	284
Phthalic acid, di(2-propylpentyl) ester	37.56	22.30	C_24_H_38_O_4_	390
1,2-Benzenedicarboxylic acid	37.93	63.26	C_24_H_38_O_4_	390

* Note: Retention time (RT); molecular formula (MF); molecular weight (MW).

**Table 3:** GC-MS analysis of metabolized medium extract of *A. niger*.

Detected Constituent	RT *	Area (%)	MF *	MW *
Acetic acid ethyl ester	5.08	0.71	C_4_H_8_O_2_	88
*N*-(4,6-Dimethyl-2-pyrimidinyl)-4-(4-nitrobenzylideneamino) benzenesulfonamide	14.47	4.61	C_19_H_17_N_5_O_4_S	411
Pentadecanoic acid, 14-methyl-, methyl ester	26.82	1.42	C_17_H_34_O_2_	270
Hexadecanoic acid	28.78	7.42	C_16_H_32_O_2_	256
9,12-Octadecadienoic acid, methyl ester, (E,E)-	29.99	5.21	C_19_H_34_O_2_	294
9-Octadecenoic acid (Z)-	31.73	3.64	C_18_H_34_O_2_	282
Octadecanoic acid	32.23	2.49	C_18_H_36_O_2_	284
Phthalic acid, di(2-propylpentyl) ester	37.56	22.49	C_24_H_38_O_4_	390
Diisooctyl phthalate	37.72	0.15	C_24_H_38_O_4_	390
1,2-Benzenedicarboxylic acid	37.93	51.87	C_24_H_38_O_4_	390

* Note: Retention time (RT); molecular formula (MF); molecular weight (MW).

**Table 4:** Antimicrobial activity of *P. rubens*, *A. alternata* and *A. niger*.

Test Organism	Inhibition Zone (mm)				MIC of *P. rubens* Filtrate Extract µg/mL
	*P. rubens*	*A. alternata*	*A. niger*	Control *	
*B. subtilis*	27.20	17.75	20.15	22.33	19.50
*S. aureus*	22.21	14.20	17.10	20.20	22.30
*E. coli*	26.26	21.05	21.50	24.43	21.62
*P. aeruginosa*	27.33	22.20	16.25	24.42	19.70
*C. albicans*	28.25	21.15	19.33	24.50	31.2
*A. fumigatus*	8.50	0.00	0.00	6.20	39.50

Control *, loaded discs with gentamycin as antibacterial agent and ketoconazole as antifungal agent.

**Table 5:** Docking scores and energies of *N*-(4,6-dimethyl-2-pyrimidinyl)-4-(4-nitrobenzylideneamino) benzenesulfonamide and 1,2-benzenedicarboxylic acid with 6TZ6 receptors.

Mol	mseq	S	rmsd_Refine	E_Conf	E_Place	E_Score1	E_Refine	E_Score2
*N*-(4,6-Dimethyl-2-pyrimidinyl)-4-(4-nitrobenzylideneamino) benzenesulfonamide	1	−6.04905	0.6673	−69.0445	−50.0656	−9.10488	−25.0128	−6.04905
	1	−5.91481	1.6653	−67.9808	−66.4094	−7.9697	−30.5244	−5.91481
	1	−5.82162	1.5377	−78.9276	−75.9068	−8.11409	−30.4167	−5.82162
	1	−5.81604	2.2649	−68.0293	−45.1237	−7.79368	−27.426	−5.81604
	1	−5.73762	1.5874	−77.267	−44.5125	−8.87414	−31.9882	−5.73762
1,2-Benzenedicarboxylic acid	2	−4.74927	0.6303	−126.93	−39.1911	−8.92265	−13.603	−4.74927
	2	−4.45324	0.4808	−130.454	−60.5226	−8.77861	−16.4348	−4.45324
	2	−4.40914	0.6678	−126.281	−48.3632	−9.36907	−11.4361	−4.40914
	2	−4.35465	1.1219	−127.282	−37.8587	−8.83715	−16.659	−4.35465
	2	−4.30812	0.992	−129.415	−40.7101	−8.7569	−16.2546	−4.30812

**Table 6:** Docking scores and energies of *N*-(4,6-dimethyl-2-pyrimidinyl)-4-(4-nitrobenzylideneamino) benzenesulfonamide and 1,2-benzenedicarboxylic acid with 7CKQ receptors.

Mol	mseq	S	rmsd_Refine	E_Conf	E_Place	E_Score1	E_Refine	E_Score2
*N*-(4,6-Dimethyl-2-pyrimidinyl)-4-(4-nitrobenzylideneamino) benzenesulfonamide	1	−6.59009	1.1327	−77.8904	−60.5493	−10.7919	−34.7198	−6.59009
	1	−6.53716	1.2057	−68.1997	−72.8364	−10.2792	−33.0921	−6.53716
	1	−6.51062	4.7166	−74.721	−74.2806	−9.3112	−35.0797	−6.51062
	1	−6.17644	2.6662	−66.5713	−69.706	−9.98946	−32.9953	−6.17644
	1	−6.06362	1.9137	−80.531	−63.459	−9.48588	−31.6404	−6.06362
1,2-Benzenedicarboxylic acid	2	−3.97767	0.9546	−127.43	−45.723	−9.74105	−14.5658	−3.97767
	2	−3.94882	3.0570	−130.836	−39.1438	−8.63553	−15.1371	−3.94882
	2	−3.85854	1.5905	−130.089	−49.7431	−9.35678	−13.6882	−3.85854
	2	−3.83116	1.5718	−130.57	−45.1682	−8.80624	−13.9746	−3.83116
	2	−3.67637	1.1142	−131.241	−36.3385	−8.59382	−14.4134	−3.67637

**Table 7:** *N*-(4,6-dimethyl-2-pyrimidinyl)-4-(4-nitrobenzylideneamino) benzenesulfonamide and 1,2-benzenedicarboxylic acid interaction with 6TZ6 protein.

Mol	Ligand	Receptor	Interaction	Distance	E (kcal/mol)
*N*-(4,6-Dimethyl-2-pyrimidinyl)-4-(4-nitrobenzylideneamino) benzenesulfonamide	6-ring	N HIS 388 (A)	Pi-H	4.57	−0.9
	6-ring	CA PRO 393 (A)	Pi-H	3.55	−0.6
1,2-Benzenedicarboxylic acid	O 14	N SER 173 (A)	H-acceptor	2.91	−3.5
	O 16	OG SER 173 (A)	H-acceptor	2.81	−3.1
	6-ring	CA GLY 172 (A)	Pi-H	3.89	−0.5

**Table 8:** *N*-(4,6-dimethyl-2-pyrimidinyl)-4-(4-nitrobenzylideneamino) benzenesulfonamide and 1,2-benzenedicarboxylic acid interaction with 7CKQ protein.

Mol	Ligand	Receptor	Interaction	Distance	E (kcal/mol)
*N*-(4,6-Dimethyl-2-pyrimidinyl)-4-(4-nitrobenzylideneamino) benzenesulfonamide	O 36	NZ LYS 83 (A)	H-acceptor	3.11	−3.6
	O 45	CE LYS 155 (A)	H-acceptor	3.39	0.9
	O 45	NE2 GLN 159 (A)	H-acceptor	2.95	−3.2
	6-ring	N ASP 167 (A)	Pi-H	4.05	−0.5
1,2-Benzenedicarboxylic acid	O 15	NE2 GLN 159 (A)	H-acceptor	3.08	−3.5
	O 16	NZ LYS 155 (A)	H-acceptor	2.98	−4.3
	6-ring	CD PRO 165 (A)	Pi-H	3.77	−1.4

## Data Availability

Available within the article.

## Abbreviations

GC-MS	Gas chromatography–mass spectrometry
RT	Retention time
MIC	Minimum inhibitory concentration
SD	Standard deviation
PC3	Prostate cancer cell
MOE	Molecular Operating Environment
RMSD	Root mean square deviation
GBVI/WSA	Generalized Born Volume Integral/Weighted Surface Area

## References

[1] Amer M.S., Abd Ellatif H.H., Hassan S.W., Aboelela G.M., Gad A.M. (2019). Characterization of some fungal strains isolated from the Eastern coast of Alexandria, Egypt, and some applications of *Penicillium crustosum*. Egypt. J. Aquat. Res..

[2] Qader M.M., Hamed A.A., Soldatou S., Abdelraof M., Elawady M.E., Hassane A.S.I., Belbahri L., Ebel R., Rateb M.E. (2021). Antimicrobial and Antibiofilm Activities of the Fungal Metabolites Isolated from the Marine Endophytes Epicoccum nigrum M13 and *Alternaria alternata* 13A. Mar. Drugs.

[3] Deng Y., Liu Y., Li J., Wang X., He S., Yan X., Shi Y., Zhang W., Ding L. (2022). Marine natural products and their synthetic analogs as promising antibiofilm agents for antibiotics discovery and development. Eur. J. Med. Chem..

[4] Al Abboud M.A., Al-Rajhi A.M., Shater AR M., Alawlaqi M.M., Mashraqi A., Selim S., Al Jaouni S.K., Ghany T.M.A. (2022). Halostability and Thermostability of Chitinase Produced by Fungi Isolated from Salt Marsh Soil in Subtropical Region of Saudi Arabia. BioResources.

[5] Pinheiro E.A., Carvalho J.M., dos Santos D.C., Feitosa Ade O., Marinho P.S., Guilhon G.M., de Souza A.D., da Silva F.M., Marinho A.M. (2013). Antibacterial activity of alkaloids produced by endophytic fungus *Aspergillus* sp. EJC08 isolated from medical plant Bauhinia guianensis. Nat. Prod. Res..

[6] Bezerra J.D., Nascimento C.C., Barbosa R., da Silva D.C., Svedese V.M., Silva-Nogueira E.B., Gomes B.S., Paiva L.M., Souza-Motta C.M. (2015). Endophytic fungi from medicinal plant *Bauhinia forficata*: Diversity and biotechnological potential. Braz. J. Microbiol..

[7] Zhou S., Wang M., Feng Q., Lin Y., Zhao H. (2016). A study on biological activity of marine fungi from different habitats in coastal regions. SpringerPlus.

[8] Hamzah T.N.T., Lee S.Y., Hidayat A., Terhem R., Faridah-Hanum I., Mohamed R. (2018). Diversity and Characterization of Endophytic Fungi Isolated from the Tropical Mangrove Species, *Rhizophora mucronata*, and Identification of Potential Antagonists Against the Soil-Borne Fungus, *Fusarium solani*. Front. Microbiol..

[9] Petit P., Lucas E.M.F., Abreu L.M., Pfenning L.H., Takahashi J.A. (2009). Novel antimicrobial secondary metabolites from a *Penicillium* sp. isolated from Brazilian cerrado soil. Electron. J. Biotechnol..

[10] Gharaei-Fathabad E., Tajick-Ghanbary M.A., Shahrokhi N. (2014). Antimicrobial Properties of *Penicillium* Species Isolated from Agricultural Soils of Northern Iran. Res. J. Toxin.

[11] Chen M., Shen Y., Lin L., Wei W., Wei D. (2022). Mn^2+^ modulates the production of mycophenolic acid in *Penicillium brevicompactum* NRRL864 via reactive oxygen species signaling and the investigation of pb-pho. Fungal Biol..

[12] Lopes F.C., Tichota D.M., Sauter I.P., Meira S.M.M., Segalin J., Rott M.B., Rios A.O., Brandelli A. (2013). Active metabolites produced by *Penicillium chrysogenum* IFL1 growing on agro-industrial residues. Ann. Microbiol..

[13] Omeike S.O., Kareem S.O., Lasisi A.A. (2019). Potential antibiotic-producing fungal strains isolated from pharmaceutical waste sludge. Beni-Suef Univ. J. Basic Appl. Sci..

[14] Visamsetti A., Ramachandran S.S., Kandasamy D. (2016). *Penicillium chrysogenum* DSOA associated with marine sponge (*Tedania anhelans*) exhibit antimycobacterial activity. Microbiol. Res..

[15] Nakashima T., Mayuzumi S., Inaba S., Park J.Y., Anzai K., Suzuki R., Kuwahara N., Utsumi N., Yokoyama F., Sato H. (2008). Production of bioactive compounds based on phylogeny in the genus *Penicillium* preserved at NBRC. Biosci. Biotechnol. Biochem..

[16] Leitao A.L. (2009). Potential of *Penicillium* species in bioremediation field. Int. J. Environ. Res. Public Health.

[17] Monika G., Punam G., Sarbjot S., Gupta G. (2010). An overview on molecular docking. Int. J. Drug Dev. Res..

[18] Dar A., Mir S. (2017). Molecular Docking: Approaches, Types, Applications and Basic Challenges. J. Anal. Bioanal. Tech..

[19] Guedes I.A., de Magalhães C.S., Dardenne L.E. (2014). Receptor–ligand molecular docking. Biophys. Rev..

[20] Kjer J., Debbab A., Aly A.H., Proksch P. (2010). Methods for isolation of marine-derived endophytic fungi and their bioactive secondary products. Nat. Protoc..

[21] Ellis M.B. (1971). Dematiaceous Hyphomycetes.

[22] Raper K.B., Fennel D.I., Robert E. (1973). The Genus Aspergillus.

[23] Domsch K.H., Gams W., Anderson T. (1980). Compendium of Soil Fungi.

[24] Rotem J. (1994). The Genus Alternaria: Biology, Epidemiology and Pathogenicity.

[25] Leslie J.F., Summerell B.A. (2006). The Fusarium, Laboratory Manual.

[26] White T.J., Bruns T., Lee S., Tayler J., Innis M.A., Gelfand D.H., Sninsky J.J., White T.J. (1990). Amplification and direct sequencing of fungal ribosomal RNA genes for polygenetics. PCR Protocols: A Guide to Methods and Applications.

[27] Abdel Ghany T.M., Ganash M., Bakri M.M., Al-Rajhi A.M.H. (2018). Molecular characterization of *Trichoderma asperellum* and lignocellulolytic activity on barley straw treated with silver nanoparticles. BioResources.

[28] Abdelghany T.M., Yahya R., Bakri M.M., Ganash M., Amin B.H., Qanash H. (2021). Effect of *Thevetia peruviana* seeds extract for microbial pathogens and cancer control. Int. J. Pharmacol..

[29] Bakri M.M., Al-Rajhi AM H., Abada E., Salem OM A., Shater A.-R., Mahmoud M.S., Abdel Ghany T.M. (2022). Mycostimulator of chitinolytic activity: Thermodynamic studies and its activity against human and food-borne microbial pathogens. BioResources.

[30] Al-Rajhi AM H., Yahya R., Abdelghany T.M., Fareid M.A., Mohamed A.M., Amin B.H., Masrahi A.S. (2022). Anticancer, anticoagulant, antioxidant and antimicrobial activities of *Thevetia peruviana* latex with molecular docking of antimicrobial and anticancer activities. Molecules.

[31] Qanash H., Yahya R., Bakri M.M., Bazaid A.S., Qanash S., Shater A.F., Abdelghany T.M. (2022). Anticancer, antioxidant, antiviral and antimicrobial activities of Kei Apple (*Dovyalis caffra*) fruit. Sci. Rep..

[32] Hamed A.A., Soldatou S., Qader M.M., Arjunan S., Miranda K.J., Casolari F., Pavesi C., Diyaolu O.A., Thissera B., Eshelli M. (2020). Screening Fungal Endophytes Derived from Under-Explored Egyptian Marine Habitats for Antimicrobial and Antioxidant Properties in Factionalised Textiles. Microorganisms.

[33] Ashok G., Senthilkumar G., Panneerselvam A. (2015). Diversity and seasonal variation of soil Fungi isolated from coastal area of Tuticorin Dt., Tamil Nadu, India. Int. J. Curr. Microbiol. Appl. Sci..

[34] Yadav M., Yadav A., Yadav J.P. (2014). In vitro antioxidant activity and total phenolic content of endophytic fungi isolated from Eugenia jambolana Lam. Asian Pac. J. Trop. Med..

[35] Atiphasaworn P., Monggoot S., Gentekaki E., Brooks S., Pripdeevech P. (2017). Antibacterial and Antioxidant Constituents of Extracts of Endophytic Fungi Isolated from *Ocimum basilicum* var. thyrsiflora Leaves. Curr. Microbiol..

[36] Danagoudar A., Joshi C., Ravi S., Rohit Kumar H., Ramesh B. (2018). Antioxidant and cytotoxic potential of endophytic fungi isolated from medicinal plant *Tragia involucrata* L.. Pharmacogn. Res..

[37] Kalpana D., Shanmugasundaram R., Mohan V. (2012). GC–MS analysis of ethanol extract of *Entada pursaetha* DC seed. Biosci. Discov..

[38] Pinto M.E.A., Araújo S.G., Morais M.I., Sá N.P., Lima C.M., Rosa C.A., Siqueira E.P., Johann S., Lima L.A.R.S. (2017). Antifungal and antioxidant activity of fatty acid methyl esters from vegetable oils. An. Acad. Bras. Ciências.

[39] Davoodbasha M., Edachery B., Nooruddin T., Lee S., Kim J. (2018). An evidence of C16 fatty acid methyl esters extracted from microalga for effective antimicrobial and antioxidant property. Microb. Pathog..

[40] Shah M.D., Iqbal M.O. (2018). Antioxidant activity, phytochemical analysis and total polyphenolics content of essential oil, methanol extract and methanol fractions from Commelinanudiflora. Int. J. Pharm. Pharm. Sci..

[41] Basheer M.A., Mekawey A.A., El-Kafrawy S.B., Abouzeid M.A. (2018). Antimicrobial Activities of Endophytic Fungi of Red Sea Aquatic Plant *Avicennia marina*. Egypt. J. Microbiol..

[42] Heydari H., Koc A., Simsek D., Gozcelioglu B., Altanlar N., Konuklugil B. (2019). Isolation, identification and bioactivity screening of turkish marine-derived fungi. Farmacia.

[43] Bladt T.T., Frisvad J.C., Knudsen P.B., Larsen T.O. (2013). Anticancer and antifungal compounds from Aspergillus, Penicillium and other filamentous fungi. Molecules.

[44] Yahya R., Al-Rajhi A.M.H., Alzaid S.Z., Al Abboud M.A., Almuhayawi M.S., Al Jaouni S.K., Selim S., Ismail K.S., Abdelghany T.M. (2022). Molecular Docking and Efficacy of *Aloe vera* Gel Based on Chitosan Nanoparticles against *Helicobacter pylori* and Its Antioxidant and Anti-Inflammatory Activities. Polymers.

[45] Al-Rajhi A.M.H., Qanash H., Almuhayawi M.S., Al Jaouni S.K., Bakri M.M., Ganash M., Salama H.M., Selim S., Abdelghany T.M. (2022). Molecular Interaction Studies and Phytochemical Characterization of *Mentha pulegium* L. Constituents with Multiple Biological Utilities as Antioxidant, Antimicrobial, Anticancer and Anti-Hemolytic Agents. Molecules.

